# Structural Compression of Convolutional Neural Networks with Applications in Interpretability

**DOI:** 10.3389/fdata.2021.704182

**Published:** 2021-08-26

**Authors:** Reza Abbasi-Asl, Bin Yu

**Affiliations:** ^1^ Department of Neurology, Department of Bioengineering and Therapeutic Sciences, University of California, San Francisco, San Francisco, CA, United States; ^2^ Weill Institute for Neuroscience, University of California, San Francisco, San Francisco, CA, United States; ^3^ Department of Electrical Engineering and Computer Sciences, University of California, Berkeley, Berkeley, CA, United States; ^4^ Department of Statistics, University of California, Berkeley, Berkeley, CA, United States

**Keywords:** convolutional neural networks, interpretation, compression, filter pruning, filter diversity

## Abstract

Deep convolutional neural networks (CNNs) have been successful in many tasks in machine vision, however, millions of weights in the form of thousands of convolutional filters in CNNs make them difficult for human interpretation or understanding in science. In this article, we introduce a greedy structural compression scheme to obtain smaller and more interpretable CNNs, while achieving close to original accuracy. The compression is based on pruning filters with the least contribution to the classification accuracy or the lowest Classification Accuracy Reduction (CAR) importance index. We demonstrate the interpretability of CAR-compressed CNNs by showing that our algorithm prunes filters with visually redundant functionalities such as color filters. These compressed networks are easier to interpret because they retain the filter diversity of uncompressed networks with an order of magnitude fewer filters. Finally, a variant of CAR is introduced to quantify the importance of each image category to each CNN filter. Specifically, the most and the least important class labels are shown to be meaningful interpretations of each filter.

## 1 Introduction

Deep convolutional neural networks (CNNs) achieve state-of-the-art performance for a wide variety of tasks in computer vision, such as image classification and segmentation (Krizhevsky et al., 2012; Long et al., 2015). The superior performance of CNNs for large training datasets has led to their ubiquity in many industrial applications and to their emerging applications in science and medicine. Thus, CNNs are widely employed in many data-driven platforms such as cellphones, smartwatches, and robots. While the huge number of weights and convolutional filters in deep CNNs is a key factor in their success, it makes them hard or impossible to interpret in general and especially for scientific and medical applications (Montavon et al., 2017; Abbasi-Asl et al., 2018). Compressing CNNs or reducing the number of weights, while keeping prediction performance, thus facilitates interpretation, and understanding in science and medicine. Moreover, compression benefits the use of CNNs in platforms with limited memory and computational power.

In this paper, interpretability is defined as the ability to explain or to present the decisions made by the model in understandable terms to a human (Doshi-Velez and Kim, 2017; Murdoch et al., 2019; Cui et al., 2020; Choi, 2020), say a biologist or a radiologist. Interpretability is typically studied from one of two perspectives. The first is the algorithmic interpretability and transparency of the learning mechanism. The other is post-hoc interpretability and explanation of the learned model using tools such as visualization. The first perspective attempts to answer the question that how the model learns and works, while the second perspective describes the predictions without explaining the learning mechanism. From the perspective of post-hoc interpretability, a CNN with fewer filters is easier to visualize and explain to human users, because CNNs are often visualized using the graphical explanation of their filters (Zeiler and Fergus, 2014). Thus to make more interpretable CNNs, a compression scheme should reduce the number of filters while keeping the model accurate (predictively). We call such schemes “structural compression”. In this paper, we argue that structurally compressed networks with fewer numbers of filters are easier to be investigated or interpreted by humans for possible domain knowledge gain.

The problem of compressing deep CNNs has been widely studied in recent literature, even though interpretability is not a motivating factor in the majority of these studies (Kamimura, 2019; Park et al., 2020; Kamimura, 2020; Kim et al., 2020; Guo et al., 2020; Wen et al., 2020; Pietron et al., 2020). In the classical approach to compression of CNNs, individual weights, and not filters, are pruned and quantized (Han et al., 2015). We call these classical compression schemes “weight compression”. Optimal brain damage (LeCun et al., 1989), optimal brain surgeon (Hassibi et al., 1993), Deep Compression (Han et al., 2015), binary neural networks (Rastegari et al., 2016; Kim and Park, 1995; Courbariaux et al., 2015), and SquuezeNet (Iandola et al., 2016) are some examples.

On the other hand, some studies have investigated pruning filters instead of weights, however, the interpretability of pruned networks has not been studied in detail (Alvarez and Salzmann, 2016; Wen et al., 2016; He et al., 2017; Liu et al., 2017; Luo et al., 2017; Li et al., 2019; Liu et al., 2019; Peng et al., 2019; You et al., 2019; Zhao et al., 2019). These studies are focused on high compression rates and low memory usage. In this paper, our goal is not to achieve state-of-the-art compression ratio or memory usage rates, but we aim to investigate the interpretability of a compressed network. However, to compare our compression ratio and computational cost to a baseline method, we chose the structural compression in He et al. (2014), Li et al. (2016). He et al. (2014), Li et al. (2016) have studied structural compression based on removing filters and introduced importance indices based on the average of incoming or outgoing weights to a filter.

Pruning activations or feature-maps to achieve faster CNNs has been also studied in Molchanov et al. (2017). Pruning activations can be viewed as removing filters in specific locations of the input, however, those filters almost always remain in other locations. Thus it rarely results in any compression of filters. On the other hand, pruning filters from the structure is equal to removing them from all the possible locations and avoiding storing them. Additionally, because of the simplified structure, filter-pruned networks are more interpretable compared to activation-pruned ones, therefore more applicable in scientific and medical domains.

Pruning a fully-trained neural network has a number of advantages over training the network from scratch with fewer filters. A difficulty in training a network from scratch is not knowing which architecture or how many filters to start with. While several hyper-parameter optimization techniques (Snoek et al., 2012; Fernando et al., 2017; Jaderberg et al., 2017; Li et al., 2017) exist, the huge numbers of possible architectures and filters would lead to a high computational cost in a combinatorial manner as in other model selection problems (Reed, 1993). Pruning provides a systematic approach to find the minimum number of filters in each layer required for accurate training. Furthermore, recent studies suggest that for large-scale CNNs, the accuracy of the pruned network is slightly higher compared to a network trained from scratch [(Li et al., 2017) for the VGG-16 network (Simonyan and Zisserman, 2014) and ResNet (He et al., 2016), (Kim et al., 2016) for AlexNet (Krizhevsky et al., 2012)]. For small-scale CNNs, it is possible to train a network from scratch that achieves the same accuracy as the pruned network even though the aforementioned computational cost is not trivial in this case. Additionally, in the majority of transfer learning applications based on well-trained CNNs, pruning algorithms achieve higher accuracies compared to training from scratch given the same architecture and number of filters (Branson et al., 2014; Molchanov et al., 2017). For example, Branson et al. (2014), showed that a pruned AlexNet gains 47% more classification accuracy in bird species categorization compared to training the network from scratch.

Our main contributions in this paper are two folds. First, we introduce a greedy structural compression scheme to prune filters in CNNs. A filter importance index is defined to be the classification accuracy reduction (CAR) (similarly RAR for regression accuracy reduction) of the network after pruning that filter. This is similar in spirit to the regression variable importance measures in Breiman (2001), Lei et al. (2018). We then iteratively prune filters in a greedy fashion based on the CAR importance index. Although achieving a state-of-the-art compression ratio is not the main goal in this paper, we show that our CAR structural compression scheme achieves higher classification accuracy in a hold-out test set compared to the baseline structural compression methods. CAR compressed AlexNet without retraining can achieve a compression ratio of 1.17 (for layer 1) to 1.5 (for layer 5) while having a close-to-original classification accuracy (54% top-1 classification accuracy compared to original 57%). This is 21% (for layer 1) to 43% (for layer 5) higher than the compression ratio from the benchmark method. If we fine-tune or retrain the CAR-compressed network, the compression ratio can be as high as 1.79 (for layer 3) when maintaining the same 54% classification accuracy. We take advantage of weight pruning, quantization, and coding by combining our method with Deep Compression (Han et al., 2015) and report a considerably improved compression ratio. For AlexNet, we reduce the size of individual convolutional layers by a factor of 8 (for layer 1) to 21 (for layer 3), while achieving close to original classification accuracy (or 54% compared to 57%) through retraining the network.

Our second contribution is bridging the compression and interpretation for CNNs. We demonstrate the ability of our CAR algorithm to remove functionally redundant filters such as color filters making the compressed CNNs more accessible to human interpreters without much classification accuracy loss. Furthermore, we introduce a variant of our CAR index that quantifies the importance of each image class to each CNN filter. This variant of our CAR importance index has been presented in Abbasi-Asl and Yu (2017) and is included in Section 4 of this paper to establish the usefulness of the CAR index. Through this metric, a meaningful interpretation of each filter can be learned from the most and the least important class labels. This new interpretation of a filter is consistent with the visualized pattern selectivity of that filter.

The rest of the paper is organized as follows. In Section 2, we introduce our CAR compression algorithm. The performance of the compression for the state-of-the-art CNNs in handwritten digit image and naturalistic image classification tasks is investigated in Section 3.1. In Section 3.2, we connect compression to the interpretation of CNNs by visualizing the functionality of pruned and kept filters in a CNN. In Section 4, a class-based interpretation of CNN filters using a variant of our CAR importance index is presented. The paper is concluded in Section 5.

## 2 CAR-Based Structural Compression

### 2.1 Notation

We first introduce notations. Let $w_{i}^{L}$ denote the *ith* convolutional filter in layer *L* of the network and *n*_*L*_ the number of filters in this layer (*i* ∈ {1, ‥, *n*_*L*_}). Each convolutional filter is a 3-dimensional tensor with the size of *n*_*L*−1_ × *f*_*L*_ × *f*_*L*_ where *f*_*L*_ × *f*_*L*_ is the size of spatial receptive field of the filter.

The activation or the feature map of filter *i* in layer *L* (*i* = 1, ‥, *n*_*L*_) is:

$$\alpha_{i}^{L}=f\left(w_{i}^{L}*P\right)$$ (1)

where *f*(⋅) is the nonlinear function in convolutional network (e.g., sigmoid or ReLU) and P denotes a block of activations from layer *L*−1 (i.e., the input to the neurons in layer *L*). The activation for the first layer could be patches of input images to the convolutional network.

Assuming network N is trained on classification task, top-1 classification accuracy of network N is defined as:

$$Acc\left(N\right)=\frac{N_{Correct}}{N_{Correct}+N_{Incorrect}}$$ (2)

where *N*_*Correct*_ and *N*_*Incorrect*_ are the number of correct and incorrect predicted classes, respectively.

In this paper, we use FLOPs to quantify the computational cost in each convolutional layer of the neural network. FLOPs for each layer of the network equal to the number of floating-point operations required in that layer to classify one image. Let’s assume $A\in R^{n_{L-1}\times k_{L-1}\times k_{L-1}}$ is the input feature map and $B\in R^{n_{L}\times k_{L}\times k_{L}}$ is the output feature map in layer *L* where *k*_*L*_ × *k*_*L*_ is the spatial size. The FLOPs for this convolutional layer equals $k_{L}^{2}n_{L}f_{L}^{2}n_{L-1}$. Additionally, the storage overhead for each convolutional layer of the network equals $4f_{L}^{2}n_{L-1}n_{L}$ bytes (Wu et al., 2016).

### 2.2 Proposed Algorithm

**FIGURE 1:**
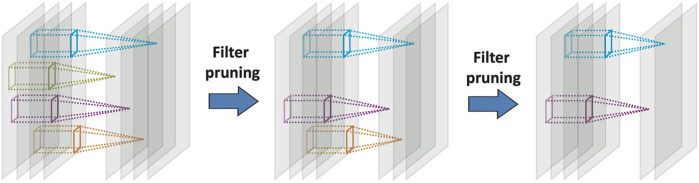
Greedy compression of CNNs based on pruning filters.

In this section, we introduce our greedy algorithm to prune filters in layers of a CNN and structurally compress it. Figure 1 shows the process of greedy filter pruning. In each iteration, a candidate filter together with its connections to the next layer gets removed from the network. The candidate filter should be selected based on an *importance* index of that filter. Therefore, defining an index of importance for a filter is necessary for any structural compression algorithm. Previous works used importance indices such as the average of incoming and outgoing weights to and from a filter but with unfortunately a considerable reduction of classification accuracy (e.g., 43% as mentioned earlier if one prunes only the first layer) for the compressed CNNs (He et al., 2014; Li et al., 2016). To overcome this limitation, we define the *importance* measure for each filter in each layer as the classification accuracy reduction (CAR) when that filter is pruned from the network. This is similar in spirit to the importance measures defined for single variables in Random Forest (Breiman, 2001) and distribution-free predictive inference (Lei et al., 2018). Formally, we define the CAR importance index for filter *i* in layer *L* of a convolutional neural network as:

CAR(i,L)=Acc(N)−Acc(N(−i,L)) (3)

where network N(−i,L) is network N except that filter *i* from layer *L* together with all of its connections to the next layer are removed from the network. In our CAR structural (or filter pruning) compression algorithm, the filter with the least effect on the classification accuracy gets pruned in each iteration. The network can be retrained in each iteration and after pruning a filter. This process is regarded as *fine tuning* in this paper. We present details of our fine-tuning procedure in the next section. Algorithm 1 shows the pseudo-code of our CAR greedy structural compression algorithm. Here, *n*_*iter*_ and *r*_*iter*_ are, respectively, the number of remaining filters and compression ratio in the current iteration.

One possible drawback of the algorithm is the expensive computational cost of the early iterations. While this is a one-time computational cost for a CNN, it is still possible to significantly reduce this cost and increase the compression speed. To accomplish this, we propose the following two simple tweaks: 1). Pruning multiple filters in each iteration of the CAR algorithm. 2). Reducing the number of images for evaluating the accuracy in each iteration (i.e., batch size). Our experiments in Supplementary Material suggest that the accuracy remains close to the original CAR compression when removing multiple filters at each iteration with smaller batch size. While these tweaks increase the compression speed, the performance of the compressed network is slightly lower than in Algorithm 1. The greedy process seems to allow for better data and network adaptation and improves compression performance. That is, when pruning one filter at each iteration, we only remove the least important filter. In the next iteration, we update all the importance indexes using the new structure. This allows the algorithm to adapt to the new structure gradually and slightly improves the classification accuracy.

The CAR compression is designed to compress each individual layer separately. This is sufficient for the majority of the transfer learning and interpretability applications because each layer is interpreted individually. However, it is possible to compress multiple layers together too (See Supplementary Material).

## 3 Results

### 3.1 Compression Rate and Classification Accuracy of the CAR Compressed Networks

To evaluate our proposed CAR structural compression algorithm, we have compressed LeNet (LeCun et al., 1998) (with two convolutional layers and 20 filters in the first layer), AlexNet (Krizhevsky et al., 2012) (with five convolutional layers and 96 filters in the first layer) and ResNet-50 (He et al., 2016) (with 50 convolutional layers and 96 filters in the first layer). LeNet is a commonly used CNN trained for classification task on MNIST (LeCun et al., 1998) consisting of 60,000 handwritten digit images. AlexNet and ResNet-50 are trained on the subset of the ImageNet dataset used in the ILSVRC 2012 competition (Russakovsky et al., 2015) consisting of more than 1 million natural images in 1,000 classes.

We used Caffe (Jia et al., 2014) to implement our compression algorithm for CNNs and fine-tune them. The pre-trained LeNet and AlexNet are obtained from the Caffe model zoo. All computations were performed on an NVIDIA Tesla K80 GPU. The CAR index is computed using half of the ImageNet test set. To avoid overfitting, the final performance of the CAR compressed network is evaluated on the other half of the ImageNet test set. The running time of each pruning iteration depends on the number of filters remaining in the layer. On average, each iteration of CAR takes 45 min for the first layer of AlexNet. For 96 filters in this layer, the total compression time is 72 h. However, in Supplemental Materials, we show that it is possible to prune up to five filters in one iteration without loss in accuracy. This reduces the total running time of the compression to 14 h. Note that this is a one-time computational cost and much less than the time required to train AlexNet on our GPU which could take weeks.

For the fine-tuning, the learning rate has been set to 0 for the layer that is being compressed, 0.001 for the subsequent layer, and 0.0001 for all other layers. The subsequent layer is directly affected by the compressed layer, therefore, requires a higher learning rate. The network is retrained for 500 iterations. This is sufficient for the classification accuracy to converge to the final value.

#### 3.1.1 LeNet on MNIST Dataset

**FIGURE 2:**
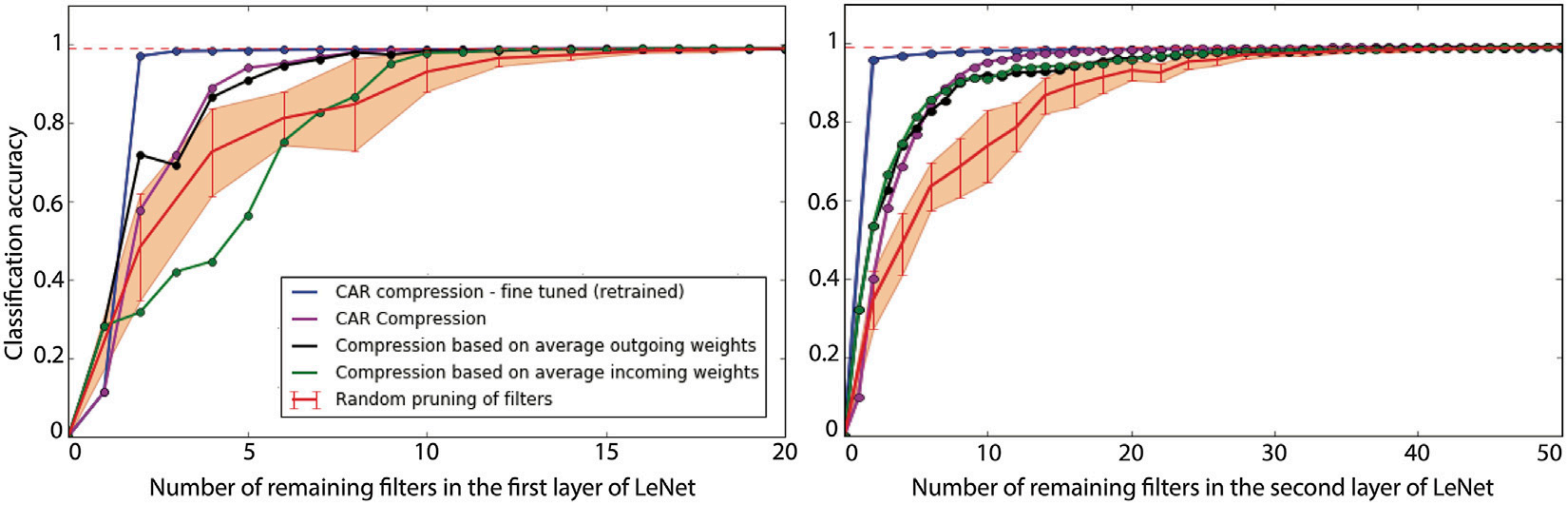
Performance of compression for LeNet. The left panel shows the overall classification accuracy of LeNet when the first convolutional layer is compressed. The right panel shows the classification accuracy when the second convolutional layer is compressed. The classification accuracy of the uncompressed network is shown with a dashed red line. The purple curve shows the classification accuracy of our proposed CAR compression algorithm for various compression ratios. The accuracy for the fine-tuned (retrained) CAR compression is shown in blue. The black and green curves show the accuracy for compressed network based on outgoing and incoming weights, respectively. The red curve shows the accuracy when filters are pruned uniformly at random. The error bar is reported over 10 repeats of this random pruning process.

LeNet-5 is a four-layer CNN consisting of two convolutional layers and two fully-connected layers. CAR-compression has been performed on the convolutional layers and the performance on a hold-out test set is reported in Figure 2. We obtained classification accuracies (top-1) of the CAR-compression results (purple curve) and those from retraining or fine-tuning after CAR-compression on the same classification task (blue curve).

To compare the performance of our compression algorithm to benchmark filter pruning schemes, we have also implemented the compression algorithm based on pruning incoming and outgoing weights proposed in He et al. (2014) and reported the classification accuracy curve in Figure 2. Furthermore, classification accuracy for random pruning of filters in LeNet has been shown in this figure. Candidate filters to prune are selected uniformly at random in this case. The error bar shows the standard deviation over 10 repeats of this random selection.

We conclude that our CAR algorithm gives a similar classification accuracy to He et al. (2014) for LeNet (using the outgoing weights in the first layer, and either weights for the second layer). Their accuracies are similar to the accuracy of the uncompressed unless we keep very few filters for either layer. Fine-tuning improves the classification accuracy but there is not a considerable gap among performances (unless we keep very few filters, less than eight among 20 for the first layer or less than 10 among 50 for the second layer). Among the eight kept filters in the first layer, four of them are shared between the CAR-algorithm and that based on averaging outgoing weights in He et al. (2014), while among the 10 kept filters in the second layer, six of them are shared.

#### 3.1.2 AlexNet on ImageNet Dataset

**FIGURE 3:**
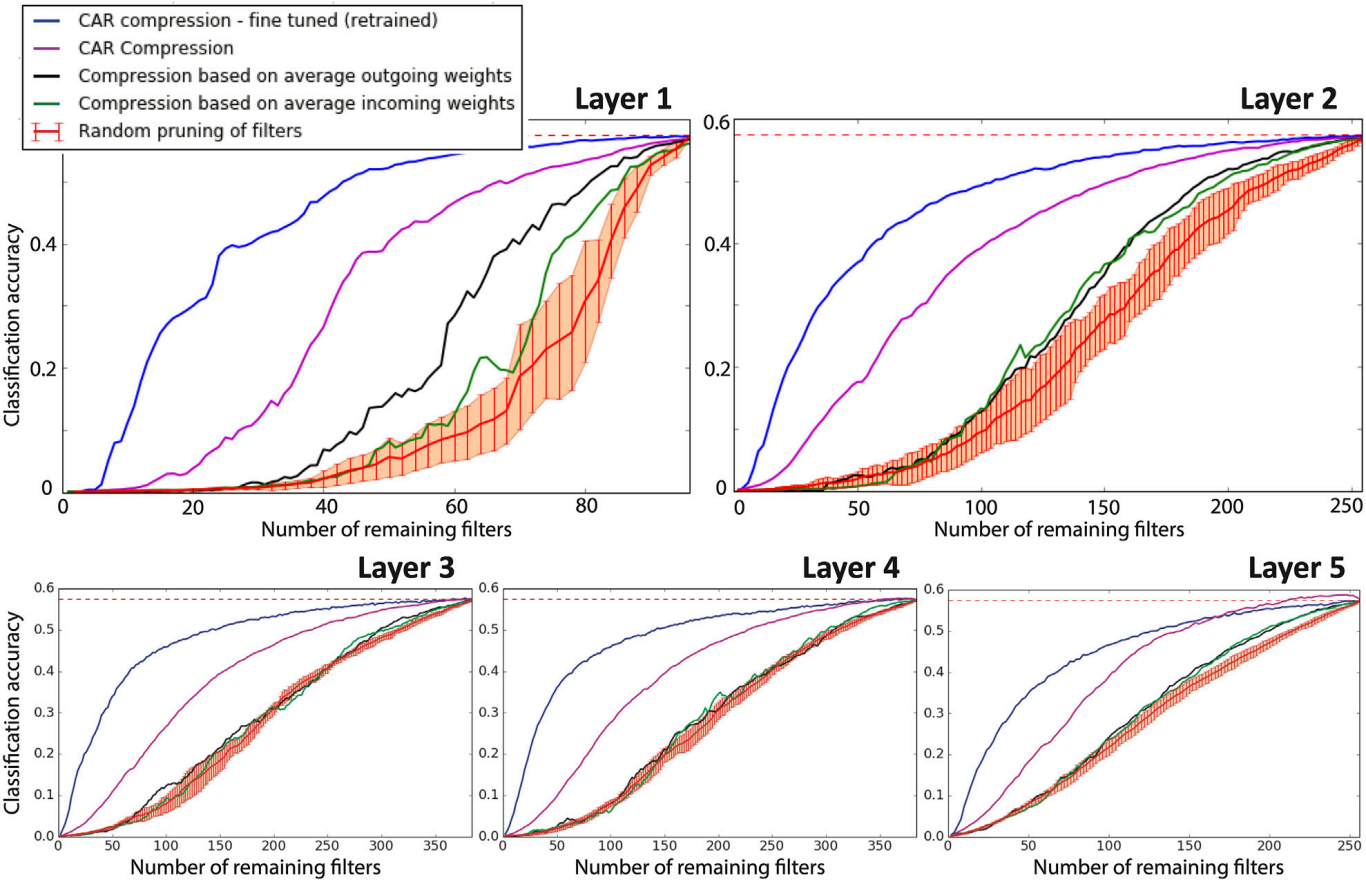
Performance of compression for AlexNet. Each panel shows the classification accuracy of the AlexNet when an individual convolutional layer is compressed. In each panel, the classification accuracy of the uncompressed network is shown with a dashed red line. The purple curve shows the classification accuracy of our proposed CAR compression algorithm for various compression ratios. The accuracy for the fine-tuned (retrained) CAR compression is shown in blue. The black and green curves show the accuracy for compressed network based on outgoing and incoming weights, respectively. The red curve shows the accuracy when filters are pruned uniformly at random. The error bar is reported over 10 repeats of this random pruning process.

AlexNet consists of five convolutional layers and three fully-connected layers. Figure 3 shows the classification accuracy of AlexNet on a hold-out test set after each individual convolutional layer is compressed using our proposed CAR algorithms or benchmark compression schemes.

Comparing the accuracies of compressed networks in Figure 3, there are considerable gaps between our proposed CAR-algorithm (purple curves) and the competing structural compression schemes that prune filters (He et al., 2014) for all five layers. Further considerable improvements are achieved by retraining or fine-tuning the CAR-compressed networks (see the blue curves in Figure 3).

Pruning half of the filters in either of the individual convolutional layers in AlexNet, our CAR algorithm achieves 16% (for layer 5) to 25% (for layer 2) higher classification accuracies compared to the best benchmark filter pruning scheme (pruning based on average outgoing weights). If we retrain the pruned network, it achieves 50–52% classification accuracy (compared with 57% of the uncompressed AlexNet). The superior performance of our algorithm for AlexNet is due to the proposed importance index for the filters in CNN. This figure demonstrates that our algorithm is able to successfully identify the least important filters for the purpose of classification accuracy. In Section 5.2, we discuss the ability of our compression scheme to reduce functional redundancy in the structure of CNNs.

To present a different but equivalent quantitative comparison, we have reported the compression ratio and feed-forward speed up in Table 1. Each individual convolutional filter is pruned while the classification accuracy dropped a relative 5% from the accuracy of the uncompressed network (i.e., 54% compared to 57%). Results for CAR compression with and without fine-tuning and compression based on average incoming and outgoing weights are presented in this table. The CAR algorithm (without retraining) can achieve a compression ratio of 1.17 (for layer 1) to 1.50 (for layer 5), which is 21–43% higher than those from the benchmark methods. If we fine-tune or retrain the CAR-compressed network, the compression ratio can be as high as 1.79 (for layer 3) when maintaining the same 54% classification accuracy.

**Table:** 

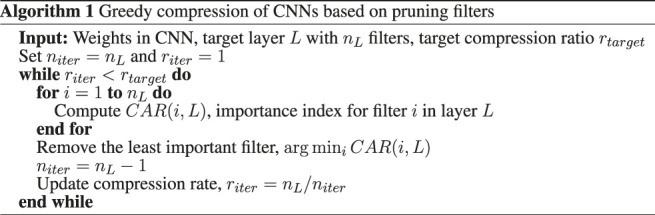

#### 3.1.3 Combination With Deep Compression

One advantage of our CAR algorithm is that it is amenable to combination with weight-based compression schemes to achieve a substantial reduction in memory usage. Deep Compression (Han et al., 2015) is a recent weight-based compression procedure that uses weight pruning, quantization, and Huffman coding. We have performed Deep Compression on top of our proposed compression algorithm and reported the compression ratio for AlexNet in Table 2. Again, the filters are pruned while the classification accuracy is in the range of relative 5% from the accuracy of the uncompressed network (54% compared to 57%). An additional five fold (for layer 1) to 12 fold (for layer 3) increase in compression ratio is achieved through joint CAR and Deep Compression. That is, further weight compression boosts the compression ratio by sparsifying weights of the kept filters, although the number of filters is the same as the CAR compression.

**TABLE 1:** Comparison of compression performance between our greedy CAR compression algorithm and benchmark schemes on AlexNet. For the compressed networks, the filters are pruned while the classification accuracy dropped a relative 5% from the accuracy of original network (i.e., 54% compared to 57%). FLOPs equals to the number of floating-point operations required in each layer to classify one image.

Layer	Compression method	Number of remaining filters	Bytes (M)	FLOPs (M)	Compression ratio and feed-forward speed up
Layer 1	Original	96	0.14	105.41	—
	Incoming weights	90	0.13	98.82	1.07×
	Outgoing weights	88	0.13	96.63	1.09×
	CAR	**82**	**0.12**	**90.04**	**1.17** **×**
Layer 2	Original	256	1.23	223.95	—
	Incoming weights	223	1.07	195.08	1.15×
	Outgoing weights	217	1.04	189.83	1.18×
	CAR	**189**	**0.91**	**165.33**	**1.35** **×**
Layer 3	Original	384	3.54	149.52	—
	Incoming weights	342	3.15	133.17	1.12×
	Outgoing weights	334	3.08	130.05	1.15×
	CAR	**287**	**2.64**	**111.75**	**1.34** **×**
Layer 4	Original	384	2.65	112.14	—
	Incoming weights	332	2.29	96.95	1.16×
	Outgoing weights	346	2.40	101.04	1.11×
	CAR	**279**	**1.93**	**81.48**	**1.38** **×**
Layer 5	Original	256	1.77	74.76	—
	Incoming weights	220	1.52	64.25	1.16×
	Outgoing weights	222	1.53	64.83	1.15×
	CAR	**171**	**1.18**	**49.94**	**1.50** **×**
Layer 1	Fine-tuned CAR	58	0.08	63.69	1.66×
Layer 2		153	0.73	133.84	1.67×
Layer 3		214	1.97	83.33	1.79×
Layer 4		225	1.56	65.71	1.71×
Layer 5		176	1.22	51.40	1.45×

The values for the best-performing models are identified in bold.

**TABLE 2:** Compression performance of CAR-algorithm combined with Deep Compression.

Layer	Weight pruning + quantization (Han et al., 2015), *Acc* = 0.57	Weight pruning + quantization + Huffman coding (Han et al., 2015), *Acc* = 0.57	CAR + weight pruning + quantization, *Acc* = 0.54	CAR + weight pruning + quantization + Huffman coding, *Acc* = 0.54
Layer 1	3.07×	4.87×	5.13×	**8.13** **×**
Layer 2	6.90×	10.60×	11.52×	**17.70** **×**
Layer 3	7.63×	11.85×	13.66×	**21.21** **×**
Layer 4	7.09×	10.98×	12.13×	**18.77** **×**
Layer 5	7.14×	10.60×	10.36×	**15.38** **×**

The values for the best-performing models are identified in bold.

#### 3.1.4 ResNet-50 on ImageNet Dataset

**FIGURE 4:**
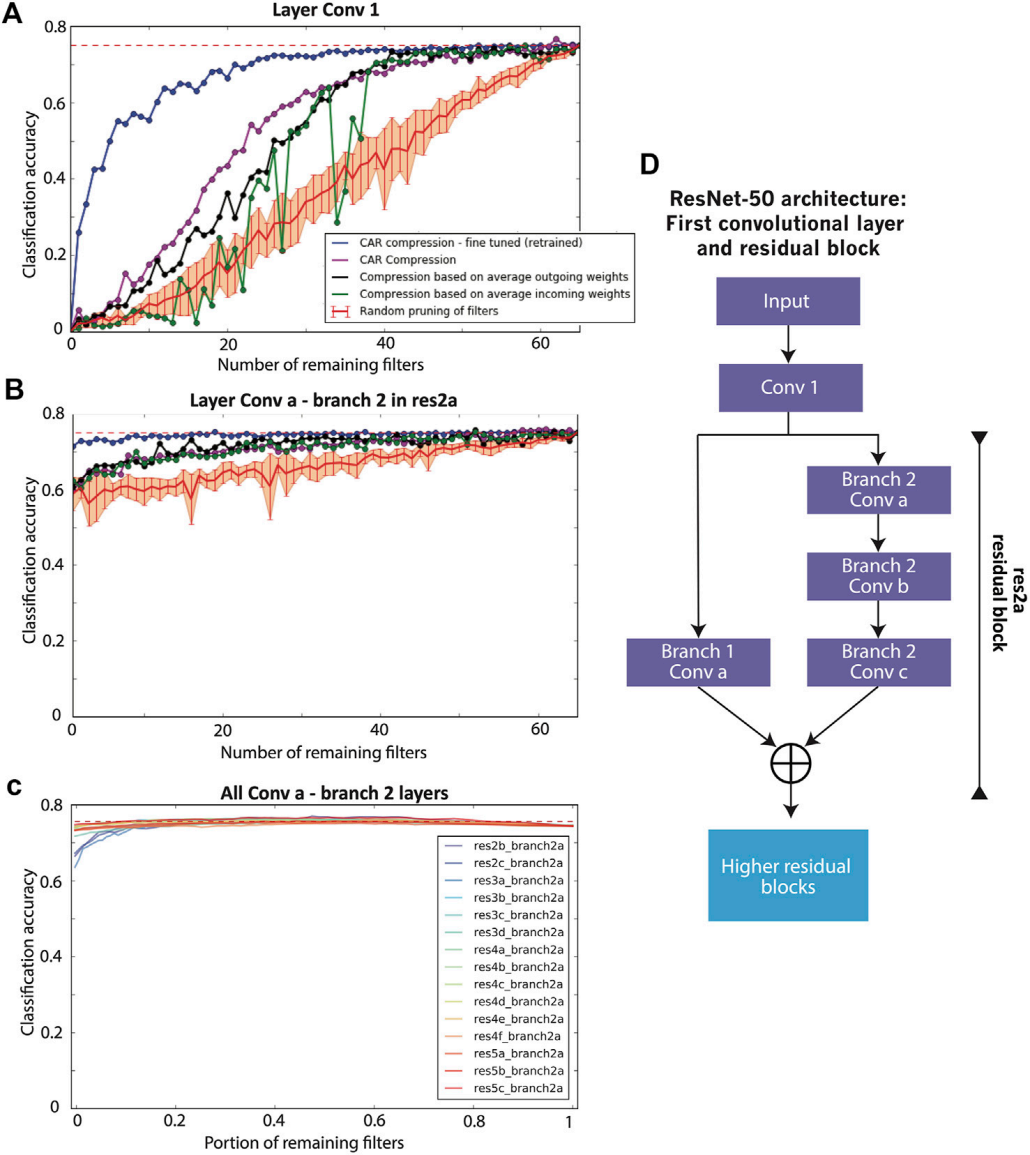
Performance of compression for ResNet-50. **(A)** Classification accuracy of the ResNet-50 for the compression of the first convolutional layer. The classification accuracy of the uncompressed network is shown with a dashed red line. The purple curve shows the classification accuracy of our proposed CAR compression algorithm for various compression ratios. The accuracy for the fine-tuned (retrained) CAR compression is shown in blue. The black and green curves show the accuracy for the compressed network based on outgoing and incoming weights, respectively. The red curve shows the accuracy when filters are pruned uniformly at random. The error bar is reported over 10 repeats of this random pruning process. **(B)** Classification accuracy for the compression of the first residual module (with the first layer untouched). **(C)** Classification accuracy for the compression of each residual module in ResNet-50. **(D)** The architecture of first layers in ResNet-50.

First introduced by He et al. (2016), deep residual networks take advantage of a residual block in their architecture (Figure 4D) to achieve higher classification accuracy compared to a simple convolutional network. We have studied the performance of CAR compression on ResNet-50 architecture (He et al., 2016) with 50 layers of convolutional weights. Figure 4A shows the classification accuracy of ResNet-50 after pruning the first convolutional layer using CAR algorithm or benchmark compression schemes. Figure 4B shows the classification accuracy after pruning the first convolutional layer in the first residual block (layer *Conv a - Branch 2* in Figure 4D). The performance for all other residual blocks is illustrated in Figure 4C. CAR pruning of other convolutional layers in each residual block yields to similar figures and is not shown here. All of the accuracies are reported on the ILSVRC 2012 ImageNet hold-out test set.

It is of great interest to compare at high compression ratio regimes where we keep less than 30 filters out of 64. In this situation and pruning layer *Conv 1*, the CAR algorithm (purple curve in Figure 4) outperforms the competitors based on incoming and outgoing weights. The higher the compression ratio, the higher the improvements by the CAR algorithm. For low compression ratio regimes, the performances are similar. Compared to AlexNet, the gap between CAR and benchmark compressions is smaller for the first layer. This might be evidence that ResNet has fewer redundant filters. Retraining (fine-tuning) the CAR-compressed network achieves further improvement in classification accuracy (blue curve in Figure 4). In fact, our CAR algorithm achieves 72% classification accuracy (compared with the 75% for the uncompressed ResNet-50) when pruning half of the filters in the first layer of ResNet-50. This accuracy is 15% higher than that of filter pruning based on average outgoing or incoming weights.

For the residual block, we have pruned layer *Conv a - Branch 2* and reported the classification accuracy in Figure 4. The accuracy of the CAR algorithm is almost similar to the compression based on incoming and outgoing weights. Interestingly, the accuracy drops less than 15% if we fully prune the filters in this layer i.e., remove branch 2 from the residual block. The drop in the accuracy is less than 5% for the fine-tuned network. The main reason for this is the existence of shortcuts in the residual module. The uncompressed branch 1 is a parallel channel with the pruned filter that allows the information to transfer through the residual layer. As a result of these parallel channels in the residual blocks, deep residual networks are more robust to pruning filters compared to simple convolutional networks.

### 3.2 CAR-Compression Algorithm Prunes Visually Redundant Filters

**FIGURE 5:**
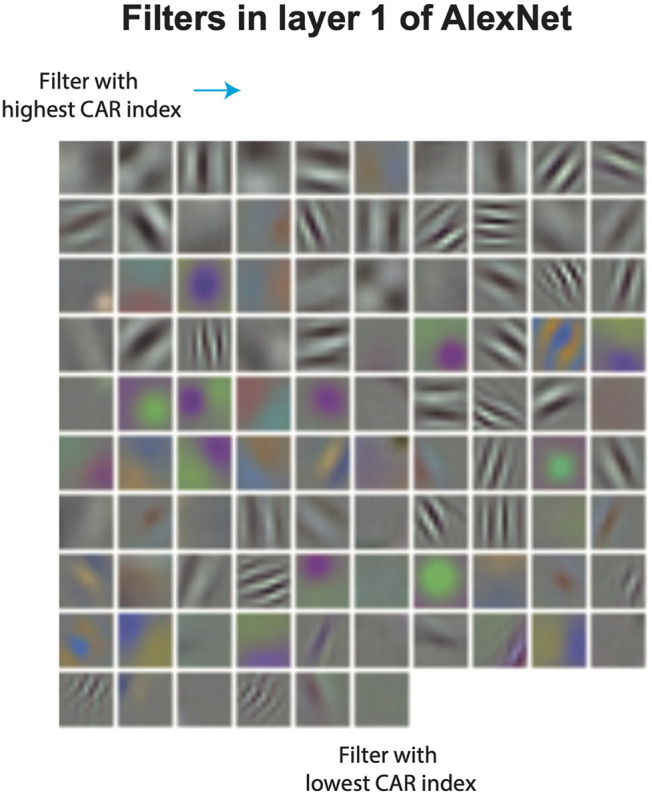
Visualization of filters in the first layer of AlexNet, ordered by their CAR importance index.

To study the ability of CAR compression in identifying redundant filters in CNNs, we take a closer look at the visualization of pruned filters. Filters in the first layer of a CNN can be visualized directly using their weights (weights in the first layer filters correspond to RGB channels of the input image). Figure 5 shows the visualized filters in the first layer of AlexNet, ordered by their CAR importance index. Filters with a higher CAR index tend to form a set of diverse patterns, spanning different orientations and spatial frequencies. Additionally, most of the filters with color selectivity tend to have a lower CAR index. In fact, out of the top 20 pruned filters, 15 of them in the first layer and 14 of them in the second layer corresponding to the color filters, respectively. This finding points to the fact that shape is often first-order important for object recognition.

**FIGURE 6:**
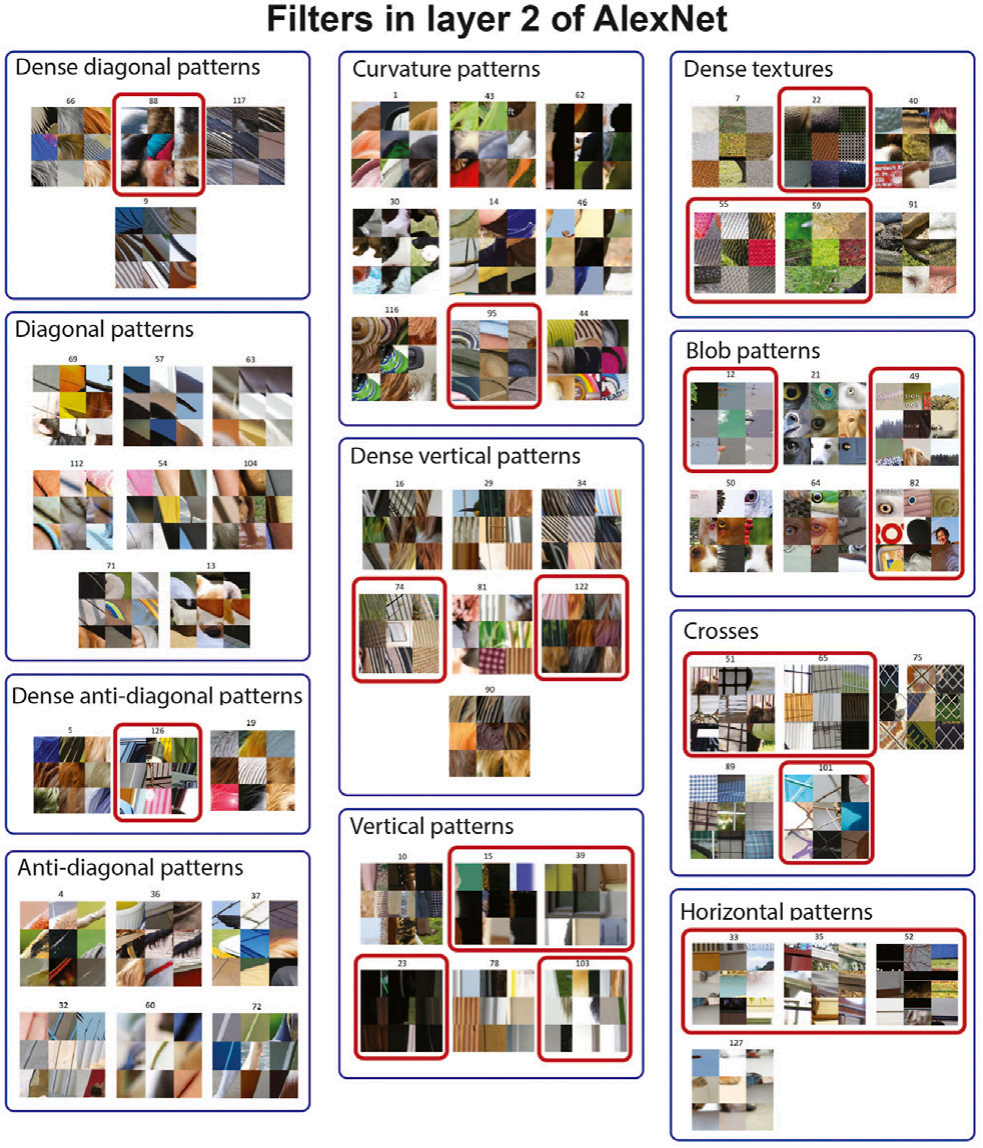
CAR compression removes filters with visually redundant functionality from the second layer of AlexNet. To visualize each filter, we have fed one million image patches to the network and visualized each filter by nine image patches with the top response for that filter. We have manually clustered 256 filters in the second layer of AlexNet into 20 clusters (11 of them visualized here) based on their pattern selectivity. We apply the CAR-based compression algorithm while the classification accuracy is in the relative range of 5% from the accuracy of the uncompressed network. This leads to pruning 103 out of 256 filters in this layer. From the 11 clusters shown in this plot, 21 filters are pruned which are identified with a red box.

Unlike the first layer, visualization of filters in higher convolutional layers is not trivial. To visualize the pattern selectivity of filters in these higher layers, we have fed one million image patches to the network and showed the top nine image patches that activate each filter. This approach has been previously used to study the functionality of filters in deep CNNs (Zeiler and Fergus, 2014). There are 256 filters in layer 2 of AlexNet which makes it challenging to visualize all of these filters. Therefore, we manually grouped filters into subsets with visually similar pattern selectivity in Figure 6. To investigate the ability of CAR compression in removing visually redundant filters in this layer, we continued to iterate the CAR algorithm while the classification accuracy is 54% or within a relative 5% from the accuracy of the uncompressed network. This led to pruning 103 filters out of 256 filters in the second layer. A subset of the removed and remaining filters are visualized in Figure 6. The filters shown in red boxes are pruned in the CAR process. Our algorithm tends to keep at least one filter from each group, suggesting that our greedy filter pruning process is able to identify redundant filters. This indicates that pruned filters based on the CAR importance index have in fact redundant functionality in the network. For some of the groups, the CAR algorithm does not select any filter to prune. For example, none of the eight filters selective for diagonal patterns or six filters selective for anti-diagonal patterns are pruned from the network. Furthermore, out of nine filters selective for curvature patterns, only one filter has been pruned. These observations suggest the importance of coding diagonal, anti-diagonal, and curvature patterns in layer 2 of the AlexNet.

**FIGURE 7:**
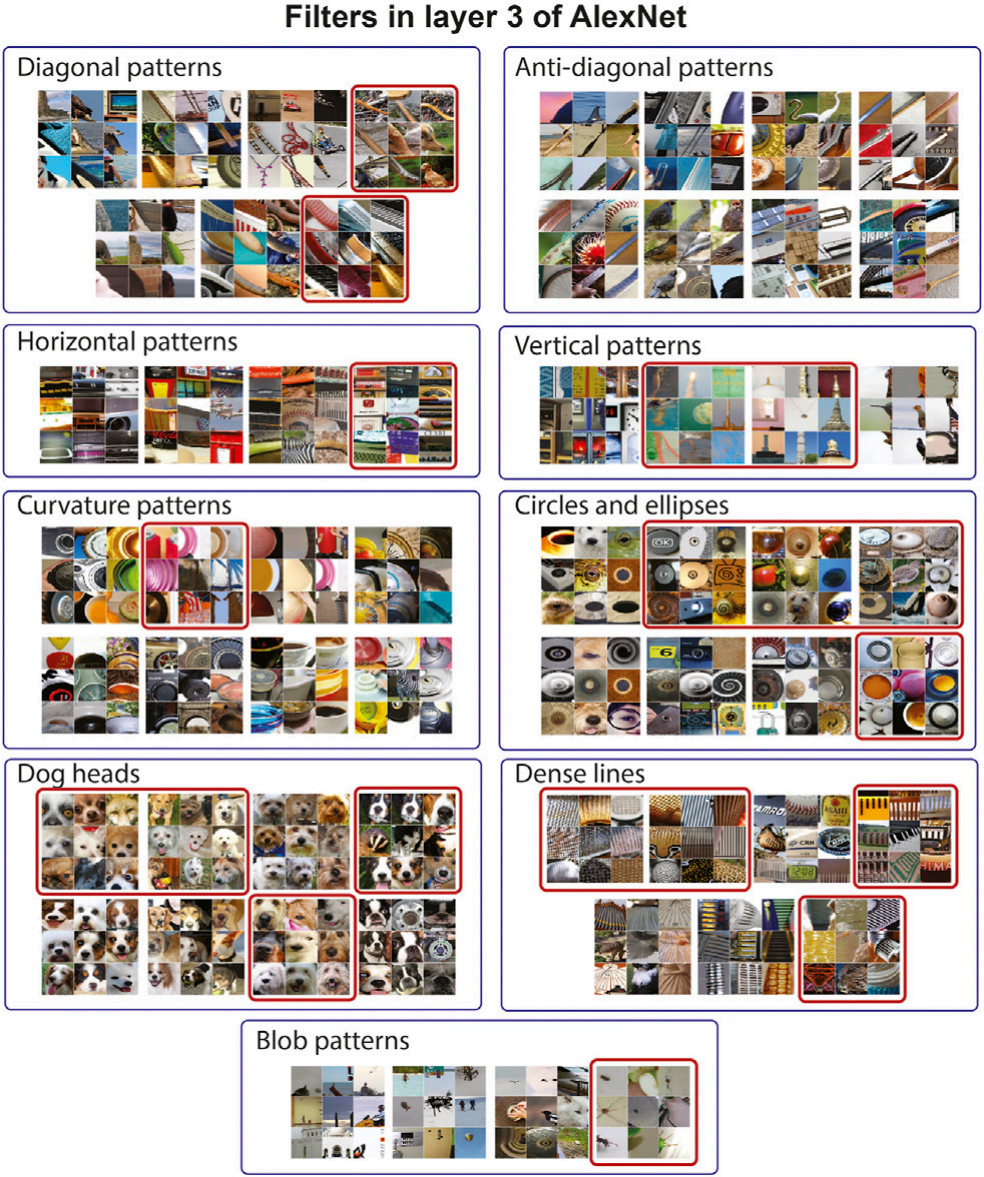
CAR compression removes filters with visually redundant functionality from the third layer of AlexNet. To visualize each filter, we have fed one million image patches to the network and visualized each filter by nine image patches with the top response for that filter. We have manually clustered filters in the third layer of AlexNet based on their pattern selectivity. nine clusters are shown in this figure with labels identifying the type of patterns. We apply the CAR-based compression while the classification accuracy is in the relative range of 5% from the accuracy of the uncompressed network. This leads to pruning 170 out of 384 filters in this layer. From the nine clusters shown in this plot, 19 filters are pruned which are identified with a red box.

The visualization of filters pruned from layer 3 of AlexNet is shown in Figure 7. Again, we performed the CAR algorithm while the classification accuracy was 54% or within a relative 5% from the accuracy of the uncompressed network. For layer 3, this resulted in pruning 170 out of 384 filters in this layer. Similar to layer 2, diagonal and anti-diagonal patterns mostly remained in the network after the pruning procedure (none of the anti-diagonal filters and 2 out of 7 diagonal filters were removed). On the other hand, 4 out of 8 filters selective to dog heads and 4 out of 8 filters selective to blobs were removed from the network which suggests the redundancy in these categories. Similar figures for layers 4 and 5 are shown in the Supplementary Material. Similar to layers 2 and 3, the CAR algorithm tends to keep at least one filter from each filter group guaranteeing the diversity of patterns encoded in each layer.

**FIGURE 8:**
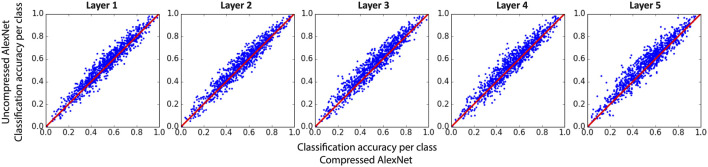
Classification accuracy for each class of image in AlexNet after the first **(left panel)** or second layer **(right panel)** is compressed compared to the uncompressed network. Each point in plots corresponds to one of the 1,000 categories of images in test set.

To further investigate the effect of compression of each of the convolutional layers, we have shown the scatter plots of the classification accuracy for each of the 1,000 classes in ImageNet in Figure 8. Although the total classification accuracy is about a relative 5% lower for each compressed network, the accuracies for many of the categories are comparable between compressed and uncompressed networks. In fact, 37% (for layer 5) to 49% (for layer 2) of the categories have accuracies no larger than 3% below those for the uncompressed network.

### 3.3 Class-Based Interpretation of Filters

With a slight modification in the definition for the CAR importance index, we build a new index to interpret the filters *via* image class labels. This index has been introduced in Abbasi-Asl and Yu (2017) and also included in this section to demonstrate the merit of the CAR index. We define *CAR* ^*c*^(*i*, *L*) to be classification accuracy reduction in class *c* of images when filter *i* in layer *L* is pruned. *CAR* ^*c*^ quantifies the importance of each filter in predicting a class label. Therefore, for each filter, we can use *CAR* ^*c*^ to identify classes that are highly dependent on that filter (classification accuracy for these classes depends on the existence of that filter). These classes are the ones with the highest *CAR* ^*c*^ among all other classes. Similarly, for each filter, the performance in classes with the lowest *CAR* ^*c*^ has less dependency on that filter.

**FIGURE 9:**
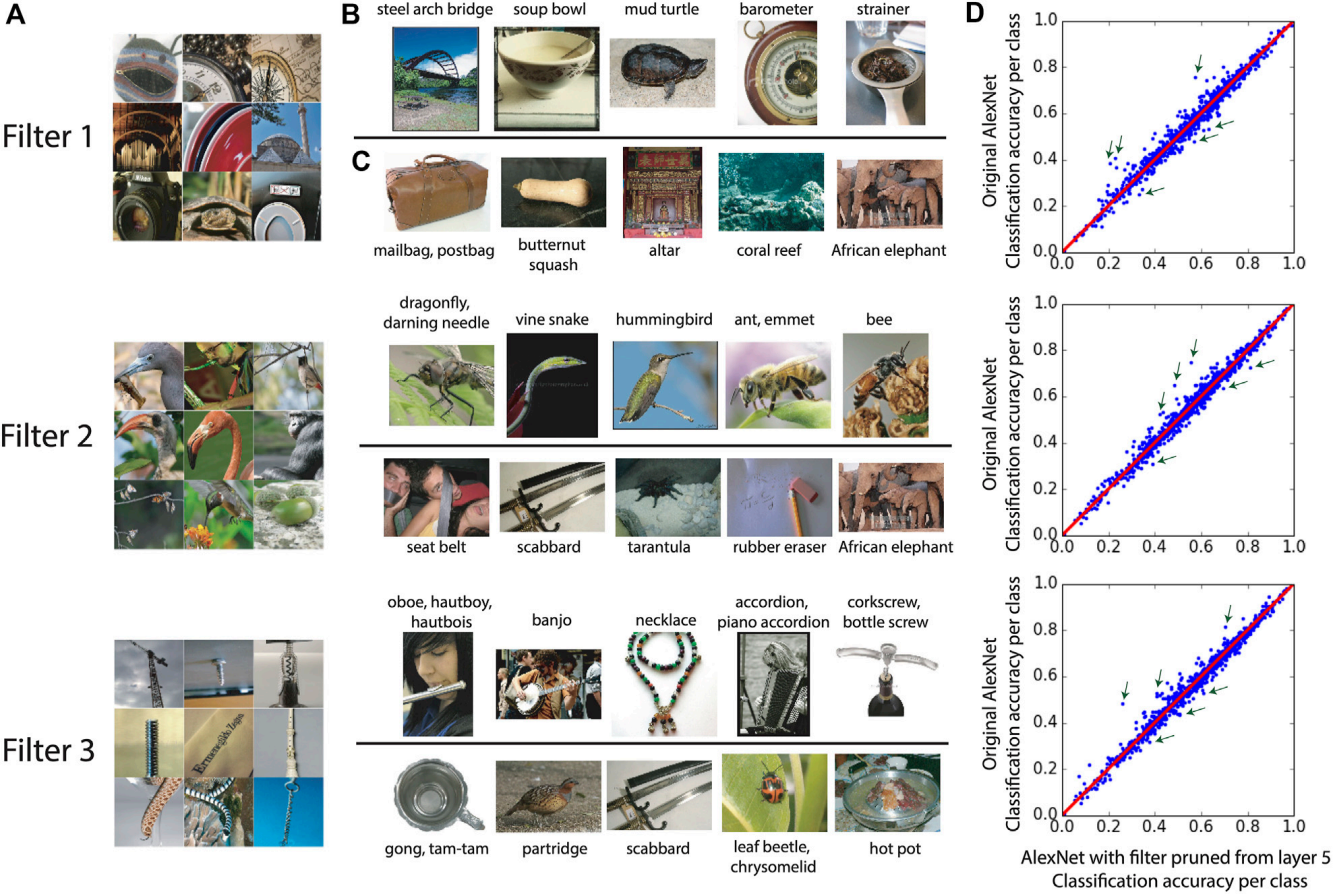
The interpretation based on *CAR* ^*c*^ is consistent with the visualized pattern selectivity of each filter in layer 5 of AlexNet. Panel **(A)** shows The top nine image patches that activate each filter (Zeiler and Fergus, 2014). Panel **(B**, **C)** show the top and bottom five classes with the highest and lowest *CAR* ^*c*^, respectively. Besides the class label, one sample image from that class is also visualized. Panel **(D)** shows the scatter plot of classification accuracy for each of the 1,000 classes in ImageNet. Three of the top and bottom classes with the highest and lowest *CAR* ^*c*^ are pointed out with green arrows. Each row corresponds to one filter in layer 5 of AlexNet.

The labels of the two sets of classes with the highest and lowest *CAR* ^*c*^ present a verbal interpretation of each filter in the network. This is particularly important in the application of CNNs in scientific domains such as medicine, where it is necessary to provide a verbal explanation of the filters for the user. *CAR* ^*c*^-based interpretation is a better fit for the higher layers in the CNN because filters in these layers are more semantic and therefore more explainable by the class labels. For these layers, the interpretation of filters based on *CAR* ^*c*^ is consistent with the visualization of pattern selectivity for that filter. Figure 9 illustrates this consistency for layer 5 of AlexNet. We focus on three filters in layer 5 that are among the most important filters in this layer based on our original CAR pruning. Similar to Figure 8, the pattern selectivity of each filter is visualized in panel A using top nine image patches activating that filter. Panels B and C show the top and bottom five classes with the highest and lowest *CAR* ^*c*^, respectively. Besides the class label, one sample image from that class is also visualized. Some of these classes are pointed out with green arrows in the scatter plot of classification accuracy for 1,000 classes in ImageNet (panel D). Note that both CAR and *CAR* ^*c*^ indexes could be negative numbers, that is the pruned network has higher classification accuracy compared to the original network.

In Figure 9, the classes with the highest *CAR* ^*c*^ share similar patterns with the top nine patches activating each filter. For filter 1, the smooth elliptic curvature that consistently appears in the classes such as *steep arch bridge* or *soup bowel* is visible in the top activating patches (Panel A). On the other hand, less elliptic curvature patterns are expected in classes such as *mailbag* or *altar*. Filter 2 has higher *CAR* ^*c*^ for classes that contain patterns such as insect or bird’s head. Filter 3 is mostly selected by the classes that contain images of a single long tool, particularly musical instruments such as *oboe* or *banjo*.

## 4 Discussion and Future Work

Structural compression (or filter pruning) of CNNs has the dual purposes of saving memory cost and computational cost on small devices, and of resulted CNNs being more humanly interpretable in general and for scientific and medical applications in particular. In this paper, we proposed a greedy filter pruning based on the importance index of classification accuracy reduction (CAR). We have shown with AlexNet that the huge gain (8 to 21 folds) in the compression ratio of CAR + Deep Compression schemes, without a serious loss of classification accuracy. Furthermore, we saw that the pruned filters have redundant functionality for the AlexNet. In particular, for many categories in ImageNet, we found that the redundant filters are color-based instead of shape-based. This suggests the first-order importance of shape for such categories.

However, a greedy algorithm is likely to be sub-optimal in identifying the best candidate filters to drop. The optimal solution may be to search through all possible subsets of filters to prune, but this can be computationally expensive and may lead to over-pruning. Procedures for subset selection, including genetic algorithms and particle swarm optimization, could be helpful in the compression of CNNs and will be investigated in future work. Even though the CAR compression of ResNet achieves state-of-the-art classification accuracy among other structural compressions by pruning the identity branch and identifying the redundant connections. ResNet compression merits further investigation because of the identity branches in the residual blocks.

We also proposed a variant of the CAR index to compare classification accuracies of original and pruned CNNs for each image class. In general, we can compare any two convolutional neural networks that are trained on a similar dataset through this index. The comparison could be done by looking into a set of classes that are important for each filter in a layer of each network. A similar class-based comparison for any two networks through our importance index is possible. This practical application of our proposed method facilitates detailed comparison of any two layers in any two networks. In fact, this is a fruitful direction to pursue, particularly given the recent wave of various CNNs with different structures. Finally, we expect that our CAR structural compression algorithm for CNNs and related interpretations can be adapted to fully-connected networks with modifications.

## Data Availability

All the data used in this study are openly available at https://image-net.org and described in Russakovsky et al. (2015).
