# Supplementary material for: Structural Compression of Convolutional Neural Networks with Applications in Interpretability
Source: Front Big Data. 2021 Aug 26;4:704182. doi: 10.3389/fdata.2021.704182 (PMC8427695; doi:10.3389/fdata.2021.704182)
Supplement: Supplementary file 1 [file DataSheet1.PDF]

Supplementary Material

1 SUPPLEMENTARY DATA

1.1 Boosting the compression speed

While CAR compression constructs a more interpretable network with sufficiently high classification performance, the main drawback is the expensive computational cost. Here, we propose two following tweaks to increase the compression speed: 1. Pruning multiple filters in each iteration of the CAR algorithm. 2. Reducing the number of images for evaluating the accuracy in each iteration (i.e. batch size). Our experiments suggest that the accuracy remains close to the original CAR compression when removing up to 5 filters at each iteration with a batch size of 128 for LeNet. Removing 5 filters at each iteration increased the computational speed by a factor of 5. Using the batch size of 128 instead of 5000 increased the speed by a factor of 12. In total, the compression speed increased by a factor of 60 for LeNet while keeping the accuracy close to the original CAR compression. Figures S1.A illustrates the classification accuracy as a function of the number of filters pruned in LeNet layer 1 when removing 1, 2, 4, or 5 filters at each iteration. The accuracy remains close to the original CAR compression when pruning 5 filters at each iteration. Panels B, C, D, and E in Figure S1 compares the accuracy curves between different batch sizes. The batch size in this figure equals the number of images from the validation set used in each iteration of CAR. For LeNet layer 1, batch size does not have a considerable effect on the curve when pruning up to 5 filters at each iteration of the algorithm. Figure S2 illustrates the accuracy curves for LeNet layer 2. Similar to layer 1, the accuracy remains close to original CAR compression when pruning up to 5 filters at each iteration with batch size 512. However, for LeNet Layer 2, a batch size of 64 degrades the accuracy.

1.2 Compressing multiple layers

To study the accuracy of CAR when multiple layers are compressed together, we used CAR to compare Figure S3 shows the classification accuracy curves as a function of the portion of remaining filters in the network. As expected, the classification accuracy decreases as we increase the number of layers involved in the compression.

1.3 CAR pruned filters for layer 4 of AlexNet

To further elaborate on the ability of CAR compression in identifying redundant filters, we have visualized filters in layer 4 of AlexNet in Figure S4. Similar to our results in the main text for layer 2, the filters are manually clustered based on their pattern selectivity. We apply the CAR pruning algorithm while the classification accuracy is in the relative range of 5% from the accuracy of the uncompressed network. The pruned filters are specified with a red box. Similar to layer 2, the CAR algorithm tends to keep filters with diverse functionality in the network.

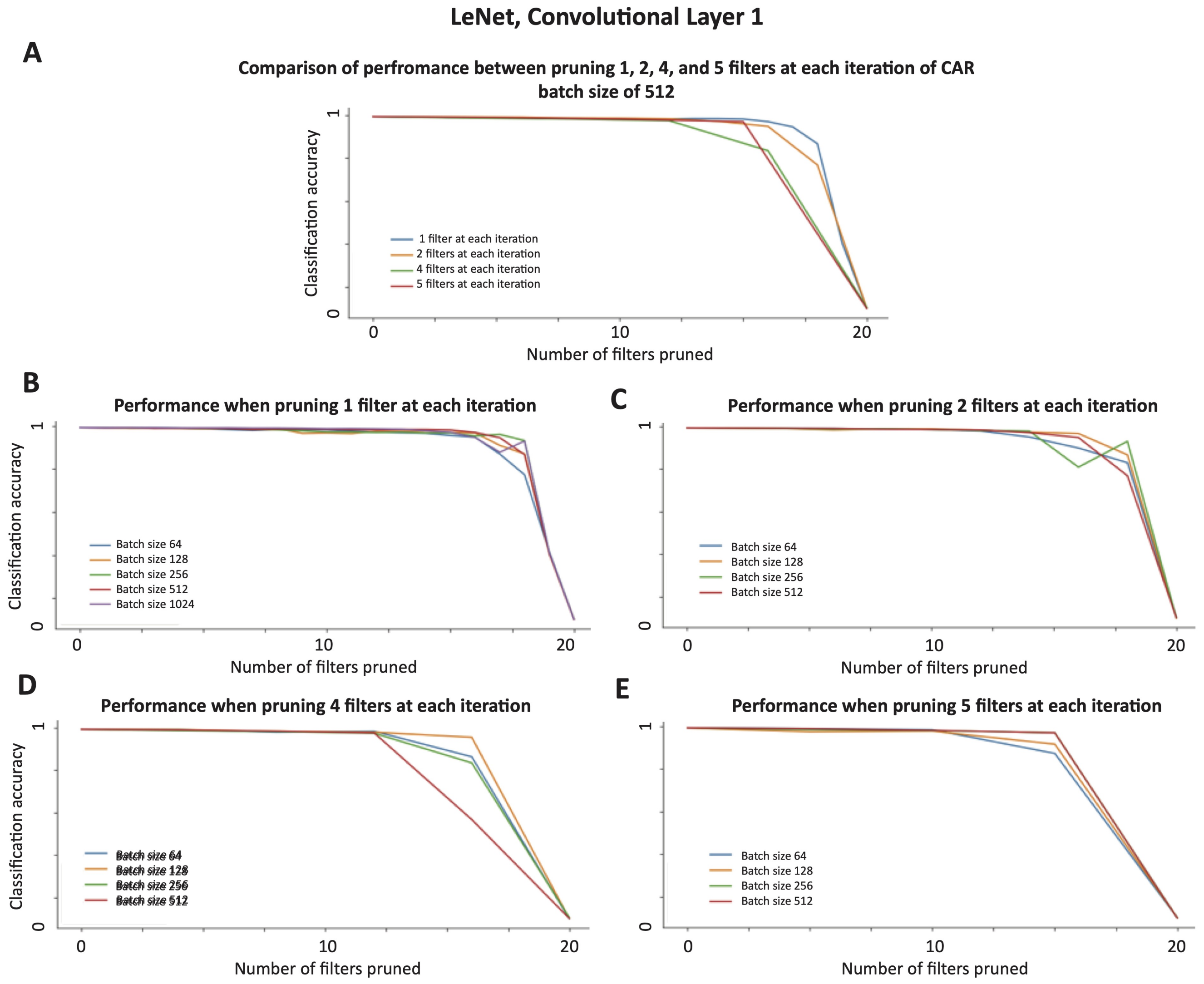

Figure S1. Removing multiple filters at each iteration of CAR algorithm boosts compression speed without degrading the accuracy for LeNet layer 1. **A** Classification accuracy as a function of the number of filters pruned in LeNet layer 1 when removing 1, 2, 4, or 5 filters at each iteration. The batch size is set to 512. The accuracy remains close to the original CAR compression when pruning 5 filters at each iteration. **B** Comparison of the accuracy curves between different batch sizes when removing 1 filter at each iteration of the algorithm. **C, D, and E** Similar to B but when removing 2, 4, or 5 filters at each iteration of the algorithm, respectively. Batch size does not have a considerable effect on the curves.

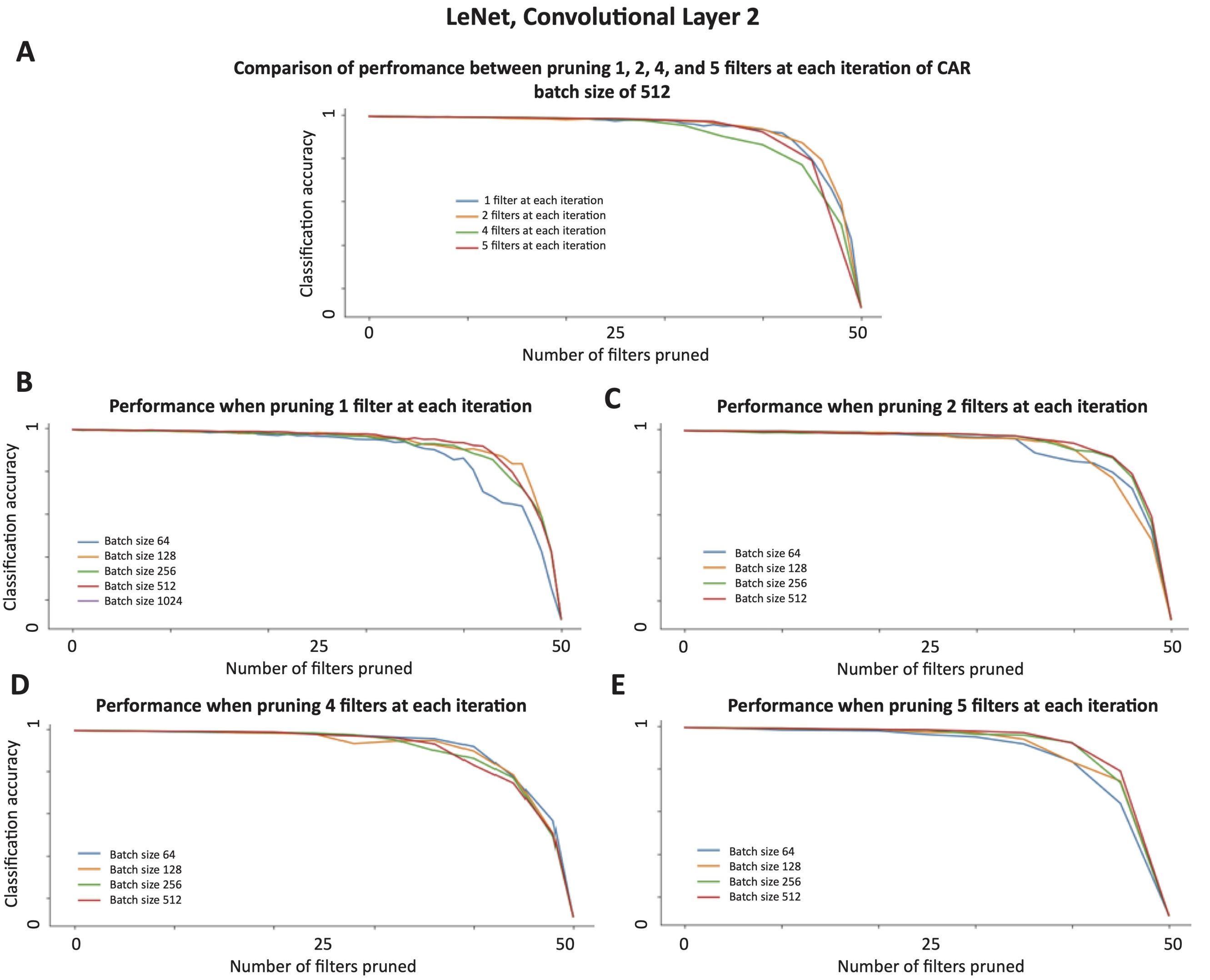

Figure S2. Removing multiple filters at each iteration of CAR algorithm boosts compression speed without degrading the accuracy for LeNet layer 2. **A** Classification accuracy as a function of the number of filters pruned in LeNet layer 2 when removing 1, 2, 4, or 5 filters at each iteration. The batch size is set to 512. The accuracy remains close to the original CAR compression when pruning 5 filters at each iteration. **B** Comparison of the accuracy curves between different batch sizes when removing 1 filter at each iteration of the algorithm. **C, D, and E** Similar to B but when removing 2, 4, or 5 filters at each iteration of the algorithm, respectively. A batch size of 64 degrades the accuracy.

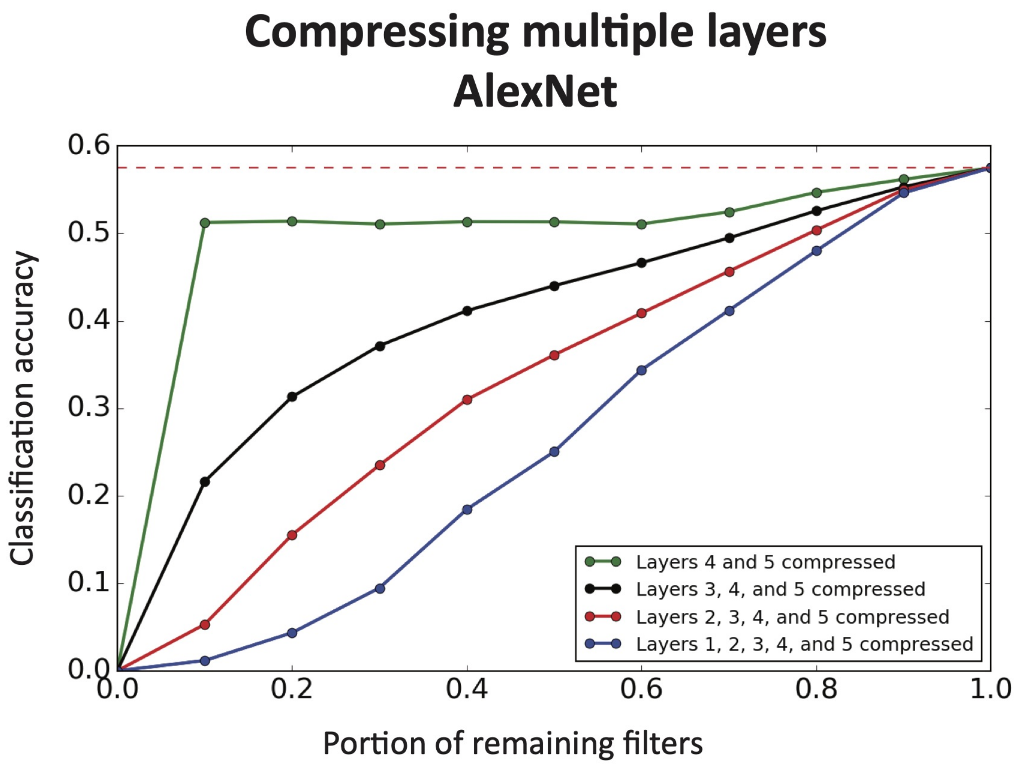

Figure S3. Classification accuracy when compressing multiple layers together. Classification accuracy curves as a function of portion of remaining filters in the network are shown for compressing multiple combination of layers. The classification accuracy decreases as we increase the number of layers involved in the compression.

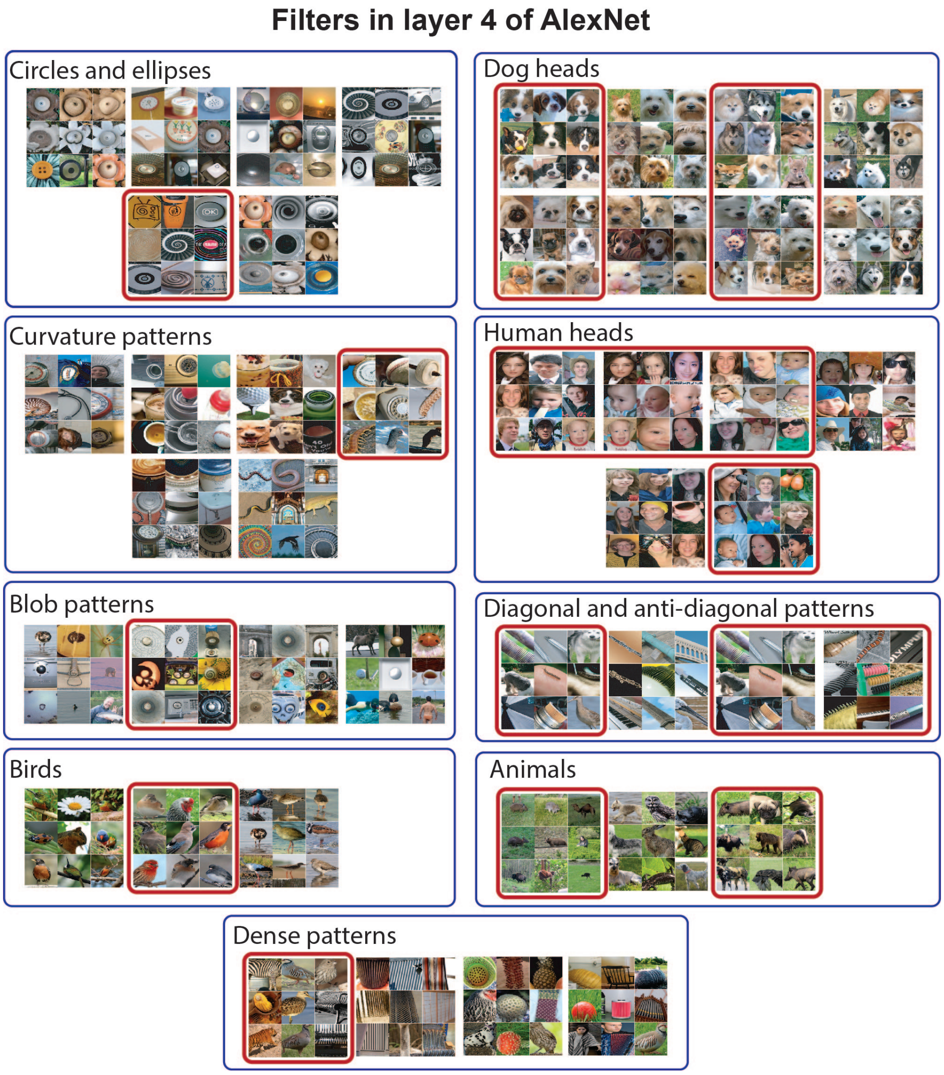

Figure S4. CAR compression removes filters with visually redundant functionality from the fourth layer of AlexNet. To visualize each filter, we have fed one million image patches to the network and visualized each filter by 9 image patches with the top response for that filter. We have manually clustered filters in the third layer of AlexNet based on their pattern selectivity. 9 clusters are shown in this figure with labels identifying the type of patterns. We apply the CAR-based compression while the classification accuracy is in the relative range of 5% from the accuracy of uncompressed network. This leads to pruning 159 out of 384 filters in this layer. From the 9 clusters shown in this plot, 18 filters are pruned which are identified with a red box.
